# CHOICES AND EXPECTATIONS OF END-OF-LIFE HEALTH CARE PROXY: A COMPARATIVE STUDY BETWEEN TAIWAN AND THE UNITED STATES

**DOI:** 10.1093/geroni/igae098.2346

**Published:** 2024-12-31

**Authors:** Duan-Rung Chen, Winston Tseng

**Affiliations:** National Taiwan University, Taipei, Taipei, Taiwan (Republic of China); University of California Berkeley, Berkeley, California, United States

## Abstract

**Objective:**

This research compares healthcare proxy preferences and expectations in Taiwan and the United States, highlighting cultural impacts on end-of-life care planning.

**Methods:**

From August 2022 to October 2023, the study used Survey Cake for online responses, employing snowball sampling. It compared 345 Taiwanese and 339 Californian respondents, matched by gender and age.

**Results:**

Despite similar demographics, distinct preferences emerged. A majority (75.5%) were under 30. In the US, 89.7% had higher education versus 75.2% in Taiwan. More Americans were married (19.2%). Preferences for healthcare proxies varied: Taiwanese favored parents, while Americans chose spouses or partners. A higher percentage of Taiwanese (61.8%) were willing and believed that their family members acting as proxies could meet their expectations, significantly higher than American respondents (48.2%). More American respondents (47.2%) expressed unwillingness to have family members make end-of-life decisions for them, contrasting sharply with Taiwanese respondents (24.2%). Regarding specific end-of-life medical decisions made by family members, Taiwanese respondents significantly favored letting healthcare proxies handle all medical decisions, including discontinuing life support, authorizing or refusing necessary pain medication or surgery among six decisions. Americans (92.7%) were more amenable to legal actions for personal wishes, markedly more than Taiwanese (66.2%).

**Conclusion:**

The study reveals significant cultural divergences between the US and Taiwan in choosing end-of-life decision-makers, reflecting deeper values and trust in the family. It underscores the importance of acknowledging cultural diversity in healthcare preferences and decisions, advocating for broader research perspectives.

